# Research in Iran: Hopes and Disappointments

**Published:** 2017

**Authors:** Shahin Akhondzadeh

In the September 15, 2016 issue of Science, Dr. Richard Stone wrote a report regarding selling theses and research articles in Iran. There are noteworthy points in this report which truly disturb the Iranian scientific community, but the present report is not the whole truth about the Iranian scientific community. Iran’s scientific infrastructure was destroyed in the eight-year imposed war by Saddam Hossein in a way that production of Iranian scientific articles in the Web of Sciences, which was twice Turkey’s scientific articles before the Islamic Revolution, decreased to one-tenth of Turkey’s scientific articles after the imposed war. Luckily, with creation of scientific infrastructure in the country’s universities in a 25-year period after the war, Iranian scientific production increased noticeably in such a way that in 2011 Iran had the greatest impetus in scientific production and also in industry, for example in production of drugs, we succeeded in producing 90% of country’s needed drugs 1,2. We succeeded in training enough physicians, dentists, and specialized physicians so that even small Iranian cities have neurosurgeons and orthopedic surgeons. Unfortunately, two problems arose in continuation of this policy:

1. Many private universities and on-line public universities began training students without logical policies especially postgraduate students without appropriate scientific infrastructure, qualified professors, and qualified students;
2. Western economic sanctions which began in 2011, although the economy was the primary focus, also targeted the Iranian scientific infrastructure. For example, university and researcher access to ISI journals via the digital library was terminated or purchase of research material such as laboratory kits for research and educational objectives encountered serious problems. Researchers and the society of Iranian scientists believe this act has not been scientific and fair. We must accept that the above mentioned two factors delivered injuries to the body of higher education. On the other hand, one must confess that at international levels, credible Iranian scientific universities move forward in education and research with power as evidenced by acceptance of Iranian medical graduates with high scores on the USMLE.

Many weak scientific journals which daily appear like mushrooms in many Southeast Asian countries and even European countries have disrupted the correct international scientific environment. The Iranian scientific society is hopeful. The Joint Comprehensive Plan of Action (JCPOA) and the vision of Dr. Rouhani’s cabinet will increase the Iranian scientific society’s endurance and will put a stop to present worries. Along the same lines, vision of the Iranian Ministry of Health and Medical Education in research evaluation of universities, in the past three years, has returned from quantity to quality. Many, in place of number of articles, put emphasis on article quality. Also, for improvement of medical university research infrastructures, a national granting body at the Health Ministry, titled NIMAD, was created which financially supports large national projects. In addition, ten large comprehensive research laboratories opened in the past three years. More than ten large cohort studies and thirty national disease registries have begun operating across the country. We are hopeful that in the next few years, exit of these large studies will result in research quality improvement in the country’s medical universities.
